# Evolving consequences of right coronary artery to right atrium: coronary cameral fistula—a case report

**DOI:** 10.1093/ehjcr/ytae207

**Published:** 2024-04-18

**Authors:** Wei Jun How, Matthew Luckie, Konstantinos Bratis, Ragheb Hasan, Nadim Malik

**Affiliations:** Manchester Heart Centre, Manchester University NHS Foundation Trust, M13 9WL, UK; Manchester Heart Centre, Manchester University NHS Foundation Trust, M13 9WL, UK; Manchester Heart Centre, Manchester University NHS Foundation Trust, M13 9WL, UK; Manchester Heart Centre, Manchester University NHS Foundation Trust, M13 9WL, UK; Manchester Heart Centre, Manchester University NHS Foundation Trust, M13 9WL, UK; Faculty of Biology, Medicine and Health, University of Manchester, M13 9PL, UK

**Keywords:** Case report, Coronary anomalies, Multimodality imaging

## Abstract

**Background:**

Coronary cameral fistula is a rare diagnosis, which may be picked up incidentally on cardiac imaging. While majority of cases is asymptomatic, they can be complicated by myocardial ischaemia, arrhythmias, heart failure, infective endocarditis, and rarely rupture or thrombosis of the fistula leading to sudden death.

**Case summary:**

A 73-year-old female presents with fever, lethargy, and examination finding of a continuous cardiac murmur. CT coronary angiogram confirmed the presence of a coronary cameral fistula, with an aneurysmal RCA seen arising from the right coronary sinus, following an extensive tortuous course wrapping around the heart, and terminating at the right atrium. While there was initial streptococcus bacteraemia identified on blood culture sampling, no obvious masses were detected on the valves, chambers, or along the course of the fistula. Over time, she develops anginal chest pain and heart failure symptoms, with progressive dilatation of the right ventricle and functional tricuspid regurgitation secondary to shunting of the fistula into the right chambers. Surgical intervention was then pursued and successfully addressed these complications.

**Discussion:**

This case report highlights the importance of advanced imaging modalities for accurate diagnosis of coronary cameral fistulae, addressing late manifestations of the disease and the necessity for a collaborative, multidisciplinary approach in managing complex cardiac anomalies.

**Learning points**

- Multimodality imaging: The case highlights the crucial role of multimodality imaging, such as CT angiography and echocardiogram, in confirming the diagnosis, assessing functional consequences of coronary cameral fistula, and guiding treatment decisions.
- Collaborative approach: Managing complex manifestation of the fistula requires close collaboration between clinical, imaging, surgical, and interventional teams to determine optimal treatment strategies and timing for intervention.

## Introduction

Coronary artery fistula (CAF) is a rare phenomenon involving anomalous termination of an artery, which often develops with an aneurysmal adaptation to a cardiac chamber or vessel. This can bypass the microcirculation, with a consequent increase in unidirectional blood flow between the connecting structures. While the majority of cases is asymptomatic and incidentally diagnosed,^1,2^ CAFs may become complicated with myocardial ischaemia, arrhythmias (such as atrial fibrillation), heart failure, infective endocarditis, and rarely rupture or thrombosis of the fistula leading to sudden death.^3^

## Summary figure

**Table:** 

Time	Events and clinical description
Index presentation	Patient presents with fever and lethargy. Noted to have audible ‘continuous’ murmur on examination. Transthoracic echocardiogram (TTE) reveals dilated right coronary artery (RCA) with turbulent flow into the right atrium. The biventricular size and systolic function on TTE were normal. Blood cultures isolated growth of *Streptococcus gordonii*. Started treatment with intravenous broad-spectrum antibiotics.
Week 1	CT coronary angiogram (CTCA) confirmed the presence of a coronary cameral fistula. An aneurysmal RCA was seen arising from the right coronary sinus, with an extensive tortuous course wrapping around the heart, and terminating at the right atrium. No obvious masses were detected on the valves, chambers, or along the course of the fistula. The patient was treated with broad-spectrum intravenous antibiotics for possible infective endocarditis.
Week 4	Discharged when clinically well and inflammatory markers have normalized.
Month 6	Presents with chest pain. Nuclear myocardial perfusion (Myoview) scan identified reversible ischaemia affecting the LAD territory. Invasive coronary angiogram showed only minimal non-obstructive coronary artery disease. Multidisciplinary discussion with surgeons ensued and no indication at present for surgical ligation. Kept under echocardiographic and clinical surveillance.
Year 3	Presents with breathlessness and ankle oedema. New atrial fibrillation diagnosed. A repeat TTE showed right ventricular dilatation and impairment with severe, functional tricuspid regurgitation, due to failure of the leaflets to coapt. The left ventricular function was shown to have deteriorated to borderline low systolic function (EF 50–54%). Due to haemodynamic impact, accepted for surgical closure/ligation of fistula with concurrent coronary artery bypass graft (saphenous venous graft to posterior descending artery), tricuspid valve repair (annuloplasty band placement), exclusion of the left atrial appendage and pericardiotomy.
Post-operative period and follow-up	Uneventful recovery. Discharged after 7-day recovery on the ward. Symptom resolution achieved with the right ventricular dimension and systolic function restored on follow-up TTE.

## Case presentation

A 73-year-old Caucasian female presented to hospital with symptoms of malaise, fever, reduced appetite, and non-specific upper abdominal discomfort. She had no significant past medical history on record. Clinical examination was essentially normal besides mild pyrexia (38°C) and an audible cardiac murmur, described to be continuous throughout the cardiac cycle. Following clinical assessment and investigations (blood tests, blood cultures, chest radiograph, and abdominal ultrasound), she was identified to have elevated inflammatory markers: white cell count 14×10^9^/L (reference range 4–11×10^9^/L), C-reactive protein level of 206 mg/L (reference range < 5 mg/L), with normal amylase levels, liver function, urea, and electrolytes. Abdominal ultrasound was normal (i.e. normal liver, gallbladder, and pancreatic appearance with no free fluid detected), and chest radiograph (CXR) indicated mild cardiomegaly only. Transthoracic echocardiogram (TTE) showed marked dilatation of the right coronary artery (RCA) with turbulent flow across into the right atrium (RA) suggestive of an RCA to RA fistula. The origin of the RCA fistula showed a holo-diastolic flow pattern demonstrated on colour flow Doppler, with a peak velocity of 1.4 m/s. In addition, there was dilatation of the coronary sinus and mild-to-moderate tricuspid regurgitation (TR) (Videos 1–3). Blood cultures isolated growth of *Streptococcus gordonii*.

**Figure 1:**
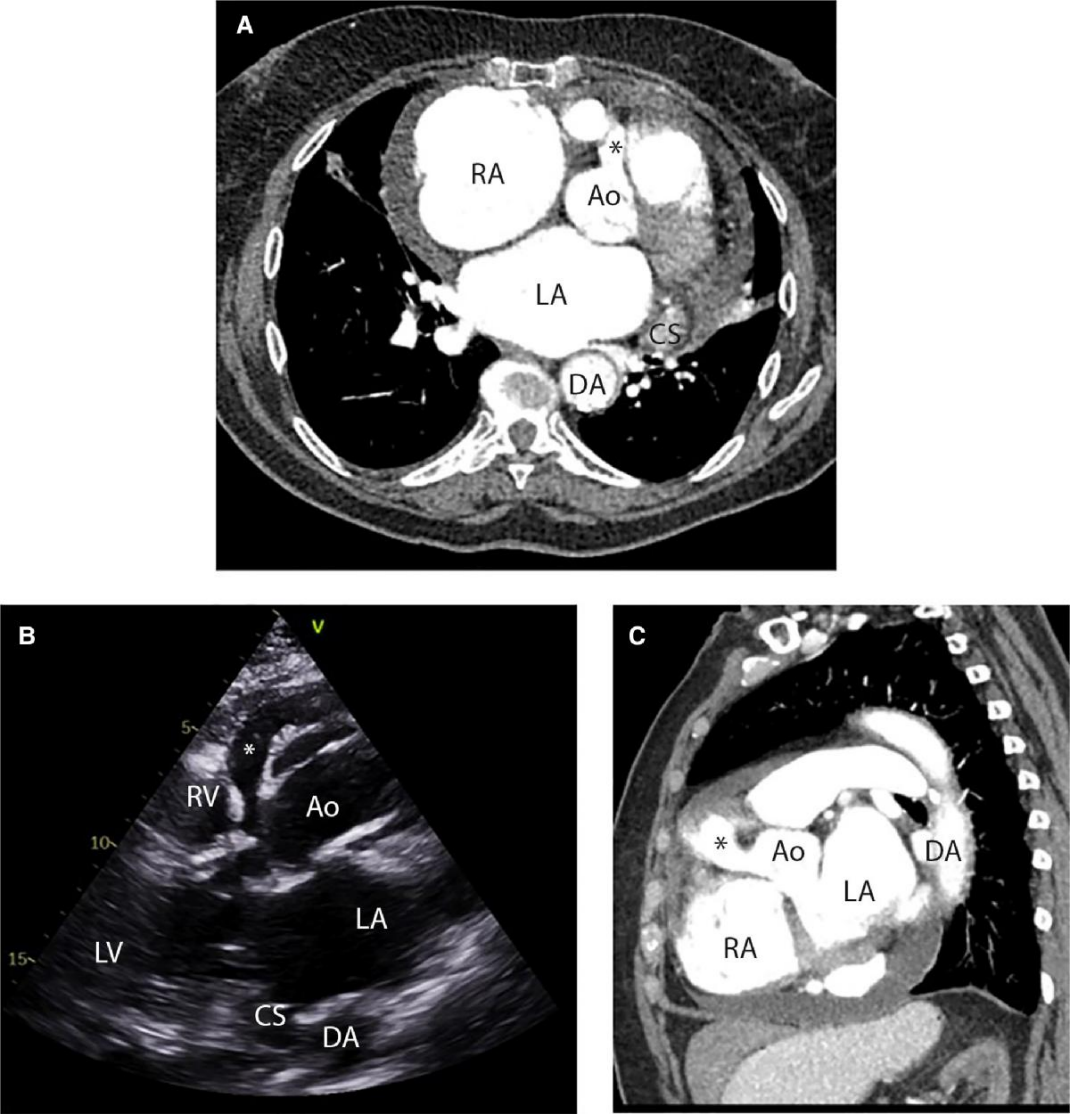
Pre-operative images demonstrating the origin of the aneurysmal right coronary artery arising from the right coronary sinus (marked with asterisk) and its relationship to adjacent structures. (*A*) Axial oblique CT, (*B*) parasternal long axis view from TTE, (*C*) sagittal oblique CT. RA, right atrium; LA, left atrium; Ao, aortic root; LV, left ventricle; RV, right ventricle; DA, descending aorta; CS, coronary sinus.

**Figure 2:**
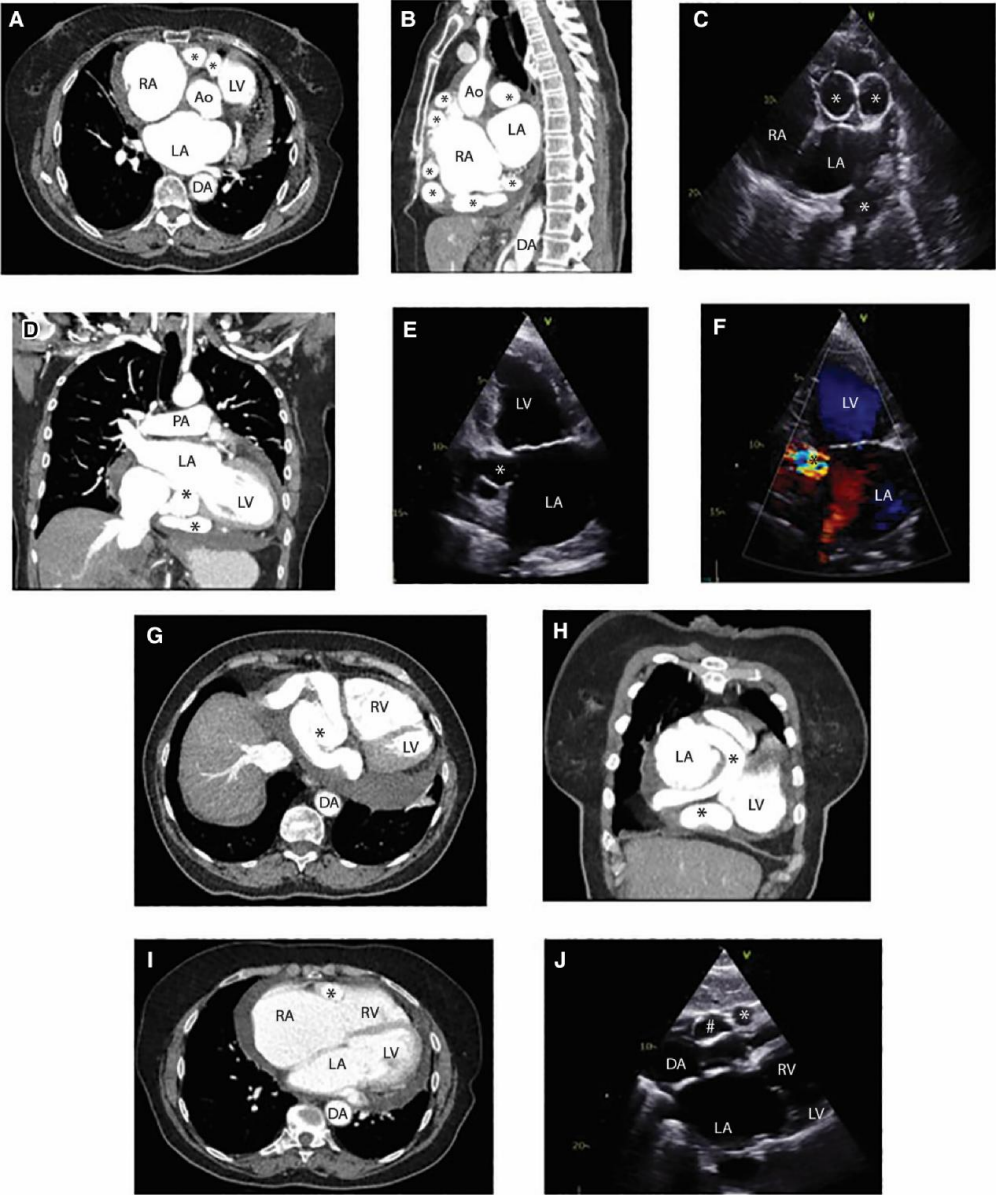
Multiple cross-sectional and transthoracic echocardiographic (TTE) images elucidating the tortuous course of the right coronary artery (RCA) (marked across all images with asterisk). (*A*, *G*, and *I*) Axial oblique slices on CT, (*B*) sagittal oblique view on CT demonstrating cross-sectional slices of the RCA ‘wrapping’ around the heart in relation to the left and right atria, (*C*) high parasternal short axis view on TTE, (*D* and *H*) coronal slices on CT, (*E* and *F*) apical two-chamber view on TTE showing the posterior course of the aneurysmal RCA with turbulent flow seen on colour Doppler. (*J*) Off axis subcostal view on TTE demonstrating the relationship of the RCA anteriorly and termination site, labelled as number sign. RA, right atrium; LA, left atrium; Ao, ascending aorta; LV, left ventricle; RV, right ventricle; DA, descending aorta; PA, pulmonary artery.

**Figure 3:**
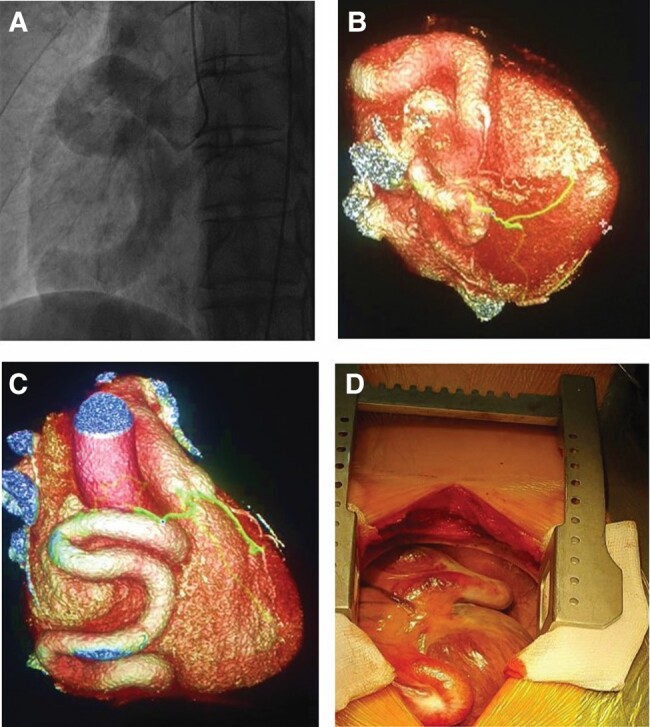
(*A*) RCA visualized on invasive coronary angiogram. (*B* and *C*) 3D reconstruction of the aneurysmal RCA on CT. (*D*) Intraoperative view of the aneurysmal RCA.

**Figure 4:**
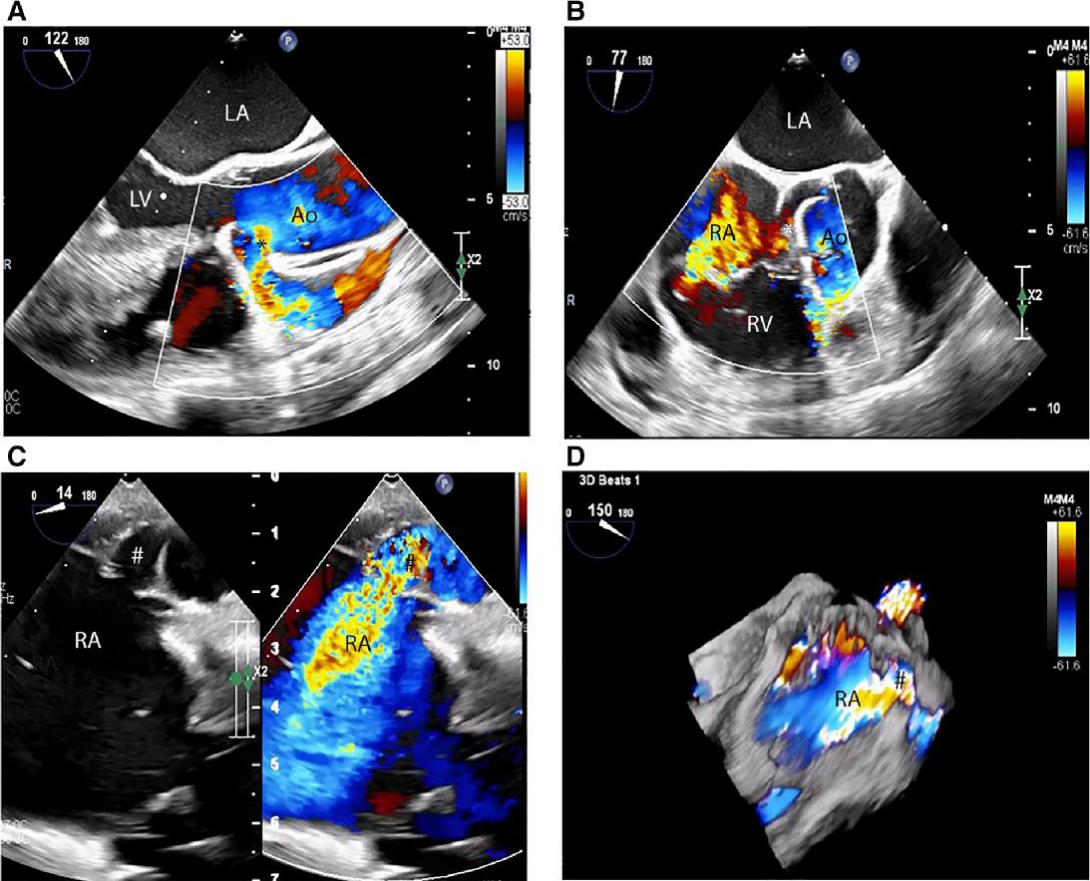
Intraoperative transoesophageal images acquired. (*A*) Mid-oesophageal long axis image demonstrating the origin and turbulent flow across the aneurysmal RCA (marked as asterisk). (*B*) Mid-oesophageal short axis image capturing both the origin of the RCA and severe jet of tricuspid regurgitation. (*C* and *D*) 2D and 3D rendered mid-oesophageal images delineating the termination site of the RCA fistula into the right atrium (marked as number sign). RA, right atrium; LA, left atrium; Ao, ascending aorta; LV, left ventricle; RV, right ventricle.

**Figure 5:**
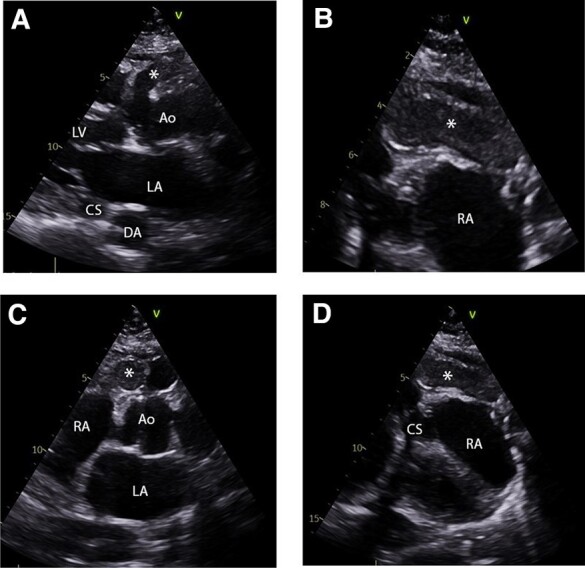
Post-operative transthoracic echocardiogram (TTE) images demonstrating thrombosed segments of the RCA (marked as asterisk) following successful closure of the fistula and concurrent saphenous venous graft (SVG) to posterior descending artery (PDA) of the RCA. RA, right atrium; LA, left atrium; Ao, ascending aorta; LV, left ventricle; DA, descending aorta; CS, coronary sinus.

In view of the initial TTE findings, cross-sectional imaging was pursued, with a CT coronary angiogram (CTCA) confirming the presence of a coronary cameral fistula. An aneurysmal RCA was observed arising from the right coronary sinus, with an extensive tortuous course wrapping around the heart, and terminating at the right atrium. The average size of the fistula measured 1 × 1 cm along its course, with dilatation of coronary sinus, which measured 0.9 × 0.8 cm at the level of the os. No obvious masses were detected on the valves, chambers, or along the course of the fistula (*Figures 1–5*). A transoesophageal echocardiogram (TOE) also confirmed these findings (Videos 4–6).

The provisional diagnosis was bacteraemia, with possible early infective endocarditis. This was presumed to be one of the late manifestations of the fistula, even in the absence of any definitive evidence available at this point to suggest that the CAF was the primary focus of infection. She was treated with a prolonged (4-week) course of parenteral broad-spectrum antibiotics (penicillin and gentamycin) as per standard local microbiology department guidelines, with an excellent response to treatment with gradual normalization of her inflammatory markers.

This patient presented few months later with new onset anginal chest pain, which was confirmed with a nuclear myocardial perfusion (Myoview) scan identifying inducible ischaemia in the left anterior (LAD) territory. A subsequent invasive coronary angiogram confirmed only non-obstructive coronary artery disease in the LAD, suitable for medical management only. She was treated with aspirin 75 mg, atorvastatin 20 mg, and bisoprolol 1.25 mg once a day. This was assumed to be another late manifestation of the fistula. Her case was discussed at a Cardiology-Cardiothoracic MDT with the consensus that in the absence of repeated infective endocarditis or haemodynamic impact secondary to the high shunted flow, surgical ligation was not indicated and therefore the patient was kept under close echocardiographic and clinical surveillance.

Three years later, nonetheless, she developed progressive breathlessness and ankle swelling. Examination revealed new atrial fibrillation (AF) with a rate of 130 beats/minute, blood pressure of 130/80 mmHg, mild pedal oedema, and normal juglar venous pressure. Auscultation confirmed a continuous murmur, known before, with new bi-basal crackles on the chest. The AF was confirmed on a 12-lead ECG and CXR identified cardiomegaly with congestive lung changes. Blood tests were normal, including serial troponins, but with elevated NT-pro-BNP levels of 5107 (reference range < 400 pg/L). A repeat TTE confirmed normal biventricular sizes and functions, with unchanged appearances of the fistula demonstrating shunted flow into the right atrium and moderate tricuspid regurgitation/TR (i.e. increased, compared to index admission) with new bi-atrial dilatation. She was commenced on standard heart failure therapy and following good response to treatment, she was discharged on apixaban 5 mg b.i.d., atorvastatin 20 mg, bisoprolol 1.25 mg, and furosemide 40 mg daily.

She underwent an elective DC cardioversion shortly after in an attempt to restore sinus rhythm but this was unsuccessful. AF-rate control strategy was then adopted, but the patient’s symptoms persisted. Another repeat TTE confirmed new right ventricular dilatation (basal diameter measurement of 48 mm and mid cavity diameter of 42 mm) and impairment with a fractional area change of 28% and severe functional TR (i.e. failure of the leaflets to coapt). The left ventricular function also deteriorated to borderline low systolic function (EF 50–54%) with a new moderate-sized pericardial effusion. Owing to the significant haemodynamic impact of the shunt and remodelling of the right ventricle, the cardiothoracic surgical team accepted her for a surgical closure/ligation of the CAF, coronary artery bypass surgery with a saphenous venous graft to the posterior descending artery of the RCA, implantation of a size 30 mm annuloplasty band to restore competence of the tricuspid valve, ligation of the left atrial appendage, and standard pericardiotomy.

Her post-operative recovery was uneventful with marked symptomatic improvement. Repeat TTE prior to discharge confirmed normal biventricular function and mild TR. The patient was discharged home after a 7-day inpatient stay. She had remained well and asymptomatic during her 3-month post-operative outpatient clinic follow-up.

## Discussion

Congenital CAF, an arterial-venous malformation, is a rare anatomical abnormality, with an approximate incidence of 0.002% in the general population. These arise from right and left coronary arteries with an even split, and terminate into lower pressure systems within the cardiac chambers or vessels in close proximity such as the coronary sinus, pulmonary artery, or superior vena cava.^2,3^ It is difficult to ascribe any specific symptoms arising from a fistula, with most cases identified due to increasing utilization of cardiac imaging modalities for general cardiac assessments.

The classical clinical sign ascribed to CAF is the presence of a continuous murmur on cardiac auscultation. Any additional clinical signs would typically incorporate the nature of presentation of the case, such as cardiac failure, cardiac dysrhythmias, or acute coronary syndrome. Non-invasive cardiac imaging with transthoracic or transoesophageal echocardiogram may identify a dilated proximal segment of the coronary artery, with ‘turbulent blood flow’ along the fistulous channel on colour flow Doppler, while detailed assessment of the origin, course, and any haemodynamic impact is achieved with advanced cross-sectional imaging modalities, CT coronary angiography (CTCA),^3^ and magnetic resonance imaging (MRI).

The present case demonstrates the utility of multimodality imaging in achieving the diagnosis of CAF, evaluating the functional consequences and need for treatment.

This coronary anomaly, when progressed, could be ascribed to a multitude of complications, including chest pain (secondary to ischaemia from any consequent steal phenomenon), breathlessness (from specific cardiac chamber enlargement/impairment leading to reduced cardiac output), palpitations (from cardiac arrhythmias with/without cardiac chamber enlargement), or rarely sudden death (from rupture of an aneurysmal segment of the artery).^4^ These fistulae may also become a nidus of infection, requiring prolonged courses of antibiotics. These complications would prompt consideration of invasive strategies such as surgical correction/ligation or transcatheter closure of the fistula.^4,5^ A multidisciplinary approach and close collaboration between the clinical, imaging, and interventional teams are paramount to guide definitive treatment and optimal timing for fistula closure or ligation.

## Supplementary Material

ytae207_Supplementary_Data

## Data Availability

The data underlying this article are available in the article and in its online Supplementary material.

## Lead author biography

Wei Jun How is a registrar/resident with dual training in General Internal Medicine and Cardiology in the UK. He is currently further subspecialising in cardiac imaging and working at the Manchester Foundation Trust.

## References

[1] Zenooz NA, Habibi R, Mammen L, Finn JP, Gilkeson RC. Coronary artery fistulas: CT findings. RadioGraphics 2009;29:781–789. 19448115 10.1148/rg.293085120

[2] Jha NK, Kiraly L, Shah N, Al Mulla A, Mora B. Congenital aneurysmal right coronary artery with a fistula to the left atrium in an adult. J Cardiothorac Surg 2019;14:33. 30736865 10.1186/s13019-019-0854-6 PMC6367818

[3] Loukas M, Germain AS, Gabriel A, John A, Tubbs RS, Spicer D. Coronary artery fistula: a review. Cardiovasc Pathol 2015;24:141–148. 25965423 10.1016/j.carpath.2014.01.010

[4] Al-Hijji M, El Sabbagh A, El Hajj S, AlKhouli M, El Sabawi B, Cabalka A, et al Coronary artery fistulas: indications, techniques, outcomes, and complications of transcatheter fistula closure. JACC: Cardiovasc Interv 2021;14:1393–1406. 34238550 10.1016/j.jcin.2021.02.044

[5] Albeyoglu S, Aldag M, Ciloglu U, Sargin M, Kematoglu T, Kutlu H, et al Coronary arteriovenous fistulas in adult patients: surgical management and outcomes. Braz J Cardiovasc Surg 2017;32:15–21. 28423125 10.21470/1678-9741-2017-0005 PMC5382904

