# Supplementary material for: Evolving consequences of right coronary artery to right atrium: coronary cameral fistula—a case report
Source: Eur Heart J Case Rep. 2024 Apr 18;8(5):ytae207. doi: 10.1093/ehjcr/ytae207 (PMC11074991; doi:10.1093/ehjcr/ytae207)
Supplement: ytae207_Supplementary_Data [file ytae207_supplementary_data.zip › Video descriptions.docx]

Video 1: Apical 4 chamber view on TTE with the fistula shunting into the right atrium. There is also associated moderate tricuspid regurgitation.

Video 2: Parasternal long axis view on TTE demonstrating marked dilatation of the right coronary artery and the origin of the coronary cameral fistula

Video 3: Subcostal view on TTE demonstrating turbulent colour flow through the coronary fistula in cross section

Video 4: Midoesophageal 4 chamber view on TOE demonstrating the shunt into the right atrium.

Video 5: 3D TOE imaging demonstrating the coronary cameral fistula and shunt into the right atrium.

Video 6:  Midoesophageal short axis view of the aortic root on TOE showing the origin of the coronary fistula arising from the right coronary cusp
